# Vitamin D Deficiency and Tuberculosis Progression

**DOI:** 10.3201/eid1605.091693

**Published:** 2010-05

**Authors:** Najeeha Talat, Sharon Perry, Julie Parsonnet, Ghaffar Dawood, Rabia Hussain

**Affiliations:** Aga Khan University, Karachi, Pakistan (N. Talat, R. Hussain); Stanford University School of Medicine, Stanford, California, USA (S. Perry, J. Parsonnet); Masoomeen General Hospital, Karachi, Pakistan (G. Dawood)

**Keywords:** Vitamin D, tuberculosis, household contacts, bacteria, Pakistan, dispatch

## Abstract

To assess the association between vitamin D deficiency and tuberculosis disease progression, we studied vitamin D levels in a cohort of tuberculosis patients and their contacts (N = 129) in Pakistan. Most (79%) persons showed deficiency. Low vitamin D levels were associated with a 5-fold increased risk for progression to tuberculosis.

Deficiency of vitamin D (25-hydroxycholecalciferol) has long been implicated in activation of tuberculosis (TB) (*1*). Serum levels of vitamin D in TB patients are lower than in healthy controls (*2*,*3*). Paradoxically, prolonged treatment of TB also causes a decline in serum vitamin D levels (*2*). Several studies have suggested that vitamin D is a potent immunomodulator of innate immune responses (*4*,*5*) by acting as a cofactor for induction of antimycobacterial activity (*6*). Of the 22 countries that have the highest TB incidence, Pakistan ranks eighth. In a previous study in Karachi, we observed that active disease developed in 7 (6.4%) of 109 TB case-contacts within 2 years (*7*). In the present study, we explored the role of vitamin D deficiency in TB disease progression within this cohort.

## The Study

Household contacts (n = 109) of 20 patients with recently diagnosed sputum-positive pulmonary TB (index case-patients) were enrolled at Masoomeen General Hospital, in Karachi during 2001–2004 for a TB household cohort study (*7*). Blood samples were collected at baseline and at 6, 12, and 24 months follow-up. Visiting health workers reviewed clinical charts every 3 months for the first 24 months and at a final home study visit during November 2007–January 2008 (45–74 months from baseline). Persons with secondary cases were referred to a consultant at Masoomeen General Hospital for additional investigation, including assessment of physical signs and symptoms, laboratory tests, chest radiographs, and sputum smear microscopy (*7*). For the present study, 129 de-identified, plasma samples preserved at –70°C from the baseline visit were shipped to Stanford University (Stanford, CA, USA) for analysis of vitamin D levels. Total circulating serum 25[OH] vitamin D was measured with ELISA by using the Immuno Diagnostic System Ltd (IDS, Fountain Hill, AZ, USA). All protocols were followed according to manufacturer’s instructions. Each test was run in duplicate, with mean absorbance computed from the average for 2 wells normalized to a zero calibrator well. Levels of vitamin D in test samples were derived by fitting a 2-parameter logistic curve to 6 standard levels and expressed as ng/mL (1 nmol/L × 0.4 = 1 ng/mL). All R^2^ values were >95%. The assay detection range was 6–360 nmol/L (2.4–144 ng/mL). Levels in 1 person were below the detection limit and were excluded from analysis. The ethical review committees of Aga Khan and Stanford universities approved the study protocol.

We used Kaplan-Meier analysis to evaluate the association of vitamin D levels with outcome of TB disease in 100 household contacts completing >1 follow-up visit. Vitamin D levels in the cohort were classified in population-based tertiles (low, middle, high). We used SAS version 9.3 (SAS Institute, Cary, NC, USA) for statistical analyses.

**Figure 1:**
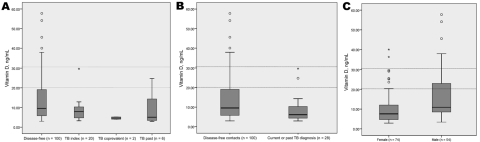
Levels of vitamin D in plasma in the Karachi, Pakistan, tuberculosis (TB) household cohort (*7*) by TB status at baseline (disease-free, index TB case-patient, coprevalent TB case-patient, and past TB case-patient, treated 2–10 years previously). One disease-free contact was excluded because of an indeterminate test result. Box plots show the median, 25th, and 75th quartiles of serum vitamin D estimated for each group (A) by any TB diagnosis (current or past) at baseline (B) and by sex (C). Reference lines represent cut-offs for insufficient and sufficient vitamin D levels, respectively. The Mann-Whitney U test was used for comparison of medians.

Median vitamin D level for the 128 cohort participants was 9.1 ng/mL (interquatrile range [IQR] 5.3–14.7); levels were 9.6 ng/mL (IQR 5.8–19.1) for 100 disease-free contacts, 7.9 ng/mL (IQR 4.7–10.3) for 20 TB index case-patients, 4.6 ng/mL (IQR 4.0–5.2) for 2 co-prevalent TB case-patients who were receiving antituberculous treatment at recruitment, and 5.1 ng/mL (IQR 3.4–14.3) in 6 household contacts with a history of TB treatment (2–10 years) (Figure 1, panel A). In the 100 disease-free household contacts, vitamin D levels were significantly higher than in the 28 participants with a history of TB diagnosis at baseline (p = 0.02; Mann-Whitney U test) (Figure 1, panel B). Median vitamin D levels were significantly lower in the 74 female patients than in the 54 male patients (7.8 vs. 11.9, Mann-Whitney U test, p = 0.0004) (Figure 1, panel C). When we stratified the cohort by vitamin D level, 79% had deficient (<20 ng/mL), 14% had insufficient (20–30 ng/mL), and 7% had sufficient (>30 ng/mL) levels of vitamin D (Table).

**Table:** Prevalence of vitamin D deficiency in a cohort study of household contacts of tuberculosis patients, Karachi, Pakistan*

Baseline characteristic	Total, no. (%)	Deficient, no. (%)	Insufficient, no. (%)	Sufficient, no. (%)	p value
Age group, y					
6–17	44 (34)	38 (86)	4 (9)	2 (5)	0.13
>18	84 (66)	63 (75)	14 (17)	7 (8)	
Sex					
Male	54 (42)	35 (65)	12 (22)	7 (13)	0.003
Female	74 (58)	66 (89)	6 (8)	2 (3)	
Tuberculin skin test					
>10 mm	101 (79)	81 (80)	14 (14)	6 (6)	0.49
<10 mm	27 (21)	20 (74)	4 (15)	3 (11)	
Total	128 (100)†	101 (79)	18 (14)	9 (7)	

*Deficient, <20 ng/mL; insufficient, 20–30 ng/mL; sufficient, >30 ng/mL. p values derived by χ^2^ test for comparison of deficient and insufficient or sufficient levels. †Excluding 1 person with indeterminate result.

**Figure 2:**
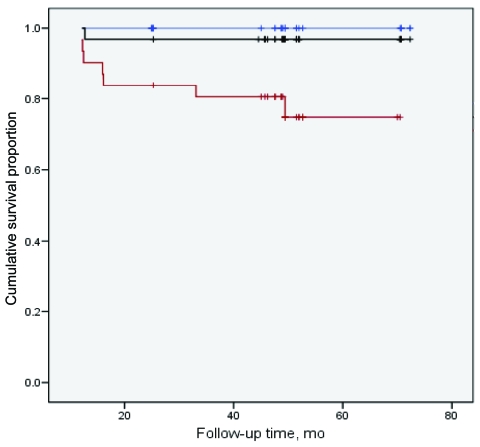
Risk for tuberculosis (TB) progression, by baseline plasma vitamin D level. Risk for progression in 100 household contacts of TB patients are indicated in cohort-based tertiles of vitamin D levels in plasma at baseline: lowest, <7.4 ng/mL (red); middle, 7.4–13 ng/mL (black); highest, >13 ng/mL (blue). Plus signs indicate censoring points. Events are defined as time to diagnosis of active TB disease during follow-up.

We next analyzed risk for progression to active TB in relation to plasma vitamin D levels. Of the 100 disease-free household contacts, 8 (8%) progressed to active disease during 4 years of follow-up. TB progression was significantly associated with relatively lower plasma vitamin D levels (Figure 2). Disease progressed in 7 (23%) of 30 patients with plasma vitamin D levels in the lowest tertile (<7 ng/mL), 1 (3%) of 32 with vitamin D levels in the middle tertile (7–13 ng/mL), and none of 30 in the highest tertile (>13 ng/mL) (p = 0.002, log rank). Six (75%) of 8 patients whose TB progressed were female patients with vitamin D levels in the lowest tertile. Further adjustment for age and sex yielded a relative risk for progression of 5.1 (1.2–21.3, p = 0.03) for a relative 1-log decrement in vitamin D levels, which suggests that vitamin D deficiency might be a strong risk factor for TB disease.

## Conclusion

In this cohort follow-up study from Pakistan, low vitamin D levels were associated with progression to active TB disease in healthy household contacts. No deaths occurred during the follow-up period from either TB or unrelated causes. Our findings also suggest that vitamin D deficiency may explain the higher susceptibility of women to disease progression in our cohort. A high prevalence of vitamin D deficiency in female patients also was reported in ambulatory patients at Aga Khan University (*8*). Factors such as low socioeconomic status, poor nutrition, traditional/cultural traits, and little exposure to sunlight may further explain vitamin D deficiency in female patients in this cohort. Despite several limitations to our study, such as information about diet, body mass index, exposure to sunlight and the relatively small number of study participants, our results are supported by a meta-analysis of 7 case-control studies in different ethnic populations (including an Indian population) that showed 70% of healthy controls had higher vitamin D levels than did untreated TB patients (*3*). Previously in African immigrants in Melbourne, Victoria, Australia (*9*), lower mean vitamin D levels were associated with high probability of latent, current, or past TB infection. Cross-sectional studies are needed in Pakistan to appreciate this association with sex and susceptibility to TB with larger sample size. Most of the South Asian population, including Pakistani immigrants to European countries and South Indians, had <10 ng/mL of serum vitamin D level (*10*) and is consistent with reports from Aga Khan Hospital (*8*,*11*). Vitamin D plays an important role in activation of 1 α-hydroxylase to convert 25(OH) D to its active form [1, 25 (OH) 2D] that leads to expression of cathelicidin, a microbicidal peptide for *Mycobacterium tuberculosis* (*5**,**12*). Serum levels >30 ng/mL provide an adequate substrate for the enzyme. Serum levels <20 ng/mL may therefore impair the macrophage-initiated innate immune response to *M. tuberculosis* and offer a possible explanation for geographic and ethnic (*13*) variations in susceptibility to TB.

Vitamin D supplementation during TB treatment remains controversial; a few studies have reported clinical improvement in pulmonary TB (*14*) and 1 study reported no effect (*15*). However, our findings indicate that further studies should be conducted regarding use of vitamin D as a supplement for persons undergoing treatment for TB and those with latent TB infection.
